# D-buddy peer support for better health outcomes in adolescents with diabetes mellitus

**DOI:** 10.1186/1687-9856-2015-S1-P15

**Published:** 2015-04-28

**Authors:** Pei Kwee Lim, Tuck Seng Cheng, Yuen Ching Angela Hui, Soo Ting Joyce Lim, Ngee Lek, Fabian Yap, Rashida Vasanwala

**Affiliations:** 1 KK Women’s & Children’s Hospital, Singapore

## Aim

Diabetes can be demanding and burdensome causing emotional distress. Non-adherence to treatment and self-care management can affect diabetes outcomes and quality of life. Peer support can play an important role in better psychological adjustment to diabetes. The aim of this study is to evaluate improvement in Quality of Life (QOL), Problem Areas in Diabetes (PAID) and glycaemic control in adolescents with diabetes

## Methods

Adolescents age 12-18 years with Type 1 or Type 2 diabetes on insulin were recruited between October 2012 to December 2013 and paired with a buddy of same age, gender and type of diabetes to provide peer support. They were instructed to contact peer buddy via telephone, SMS, Facebook, WhatsApp, or Face-to-face for 6 months. QOL, PAID scores and HbA1c were measured before and after 6 months. The adolescents who refused enrolment were treated as control group and HBA1c compared at 6 months with the study group.

## Results

A total of 66 patients (33 buddy pairs) were recruited in peer support group and 100 in control group. There were more females in peer support group (72.7%) than in the control group (49%).

In peer support group, mean number of contact episodes was 1.3 +1.4, and most common mode of contact was via SMS (43.9%), WhatsApp (27.3%) and Facebook (21.2%). There was slight improvement in mean QOL score (67.2 +14.3 vs. 69.1 +13.6; p=0.1) and also a marginal reduction in mean PAID score (25.2 +19.3 vs. 23.4 +18.8; p=0.3) indicating less negative emotions related to diabetes.

From baseline to 6 months, there was no improvement in mean HbA1c for peer support group (9.3 ±3.2 vs. 9.3 ±2.4; p=1.0) and control group (9.4 +2.2 vs. 9.3 +2.3; p=0.5).

## Conclusions

Our findings showed that there were no significant improvement in glycaemic control, quality of life and problem areas in diabetes of adolescents receiving peer support. Therefore, we need to develop other methods engaging adolescents sustain improvement in health outcomes.

